# Efficacy of Disinfectants against *Fusarium oxysporum* f. sp. *cubense* Tropical Race 4 Isolated from La Guajira, Colombia

**DOI:** 10.3390/jof7040297

**Published:** 2021-04-15

**Authors:** Luisa F. Izquierdo-García, Sandra L. Carmona, Paola Zuluaga, Gustavo Rodríguez, Miguel Dita, Mónica Betancourt, Mauricio Soto-Suárez

**Affiliations:** 1 Corporación Colombiana de Investigación Agropecuaria, AGROSAVIA. C.I Tibaitatá., Km 14 vía Mosquera-Bogotá, Cundinamarca 250047, Colombia; lfizquierdo@agrosavia.co (L.F.I.-G.); scarmona@agrosavia.co (S.L.C.); azuluaga@agrosavia.co (P.Z.); grodriguezy@agrosavia.co (G.R.); mbetancourtv@agrosavia.co (M.B.); 2 Alliance of Bioversity International and CIAT, Km 17 Recta Cali-Palmira, Cali 763537, Colombia; m.dita@cgiar.org

**Keywords:** *Fusarium* TR4, *Foc* TR4 strain 140038 isolated from La Guajira, Colombia, wilt, banana, disinfection, quaternary ammonium compounds (QACs), glutaraldehyde

## Abstract

Banana, the main export fruit for Colombia, is threatened by Fusarium wilt (FWB), caused by *Fusarium oxysporum* f. sp. *cubense* (*Foc*), tropical race 4 (TR4). Pathogen containment through disinfecting tools, machinery, shoes, and any means that may carry contaminated soil particles with proper disinfectants is at the forefront of disease management. In this study, the biocide efficacy of 10 commercial quaternary ammonium compounds (QACs) products and one based on glutaraldehyde (GA) were evaluated on both reproductive structures (microconidia and macroconidia) and survival spores (chlamydospores) of *Foc* TR4 (strain 140038) isolated from La Guajira, Colombia. QACs were evaluated at 1200 ppm and two exposure times: <1 and 15 min in the absence or presence of soil. For GA disinfectant, four different concentrations (500, 800, 1200, and 2000 ppm) were evaluated at both contact times in the presence of soil. In the absence of soil, all QACs showed 100% biocidal efficiency against microconidia, macroconidia, and chlamydospores at both <1 and 15 min. The presence of soil decreased the efficacy of disinfectants, but some of them, such as QAC3_1st, QAC7_4th, and QAC5_4th, showed 98%, 98%, and 100% efficacy against *Foc* TR4 chlamydospores, respectively, after <1 min of contact time. For instance, the GA-based disinfectant was able to eliminate all *Foc* TR4 propagules after 15 min for all concentrations tested.

## 1. Introduction

Fusarium wilt (FWB), one of the most devastating diseases of bananas, is threatening the global banana industry. The disease, caused by the soil-borne fungus *Fusarium oxysporum* f. sp. *cubense* (*Foc*), was firstly reported in Australia in 1876 [1]; however, the most destructive *Foc* TR4 strain was first detected in Taiwan in 1967 [2,3], after most likely being introduced on infected plants from Sumatra, Indonesia, and subsequently spread widely in most of the banana-producing countries [4]. The pathogen is classified into four races according to the banana cultivars they infect. Race 1 populations (R1) are pathogenic to the Gros Michel (AAA), Silk (AAB), and Pisang Awak (ABB), among other local dessert cultivars [5]. Race 2 (R2) populations affect cooking bananas, such as Bluggoe (ABB) and other edible bananas. Race 3 (R3) were populations found affecting *Heliconia* spp. but not bananas [6], so these populations are not currently considered as part of the *Foc* racial structure. Race 4 (R4) populations affect Cavendish (AAA), and also those varieties are affected by R1 and R2 populations. Race 4 was further divided into subtropical race 4 (SR4) and tropical race 4 (TR4). SR4 comprises *Foc* populations that only affect Cavendish bananas in the subtropics where FWB-predisposing conditions (such as low temperatures) might play a fundamental role [4]. TR4 refers to a clonal population that severely affects Cavendish banana (and many other cultivars) both in the tropics and the subtropics without the need for any specific temperature-related predisposing factors to cause FWB.

Tropical race 4, the most aggressive *Foc* population, was restricted to Australasia from 1990 when it was formally recognized, until 2013 when it was firstly reported in Jordan. However, in the last 8 years, new *Foc* TR4 incursions have been reported in Asia, the Middle East, the Indian subcontinent, and Africa [4,7,8,9]. In 2019, the pathogen was reported for the first time in Colombia, reaching out to the American continent [10].

The arrival of *Foc* TR4 to the American continent where seven out of the top-ten banana export countries are present raised the alarms up, not only in Colombia where the disease still has a quarantined-present with restricted distribution status but at regional levels.

*Foc* survives for long periods in the soil [5] but also in alternative hosts such as weeds [11]. The pathogen can be disseminated through propagative structures such as microconidia, macroconidia and chlamydospores [12]. Chlamydospores, which have a thickened cell wall allowing the fungi to cope with extreme environmental conditions, are considered the main survival structures. Macro- and microconidia are less resistant to unfavorable environmental conditions and play important roles on host colonization [13,14]. Both conidia and chlamydospores are produced in living plant tissues and are released to the environment once host tissue collapses [12].

As a soil-borne pathogen, any means that may carry contaminated soil can disseminate *Foc*, among them seedlings, tools, shoes, vehicles, and water. Thus, biosecurity protocols to reduce *Foc* spreading, such as disinfection of tools, working surfaces, vehicles, and equipment used in the banana plantations, need to be in place [14,15,16].

In the absence of commercially acceptable resistant varieties and effective measures to eradicate *Foc* TR4, reducing the pathogen spreading through strict containment and biosecurity measures are fundamental. Apart from developing regulatory frameworks, the effectiveness of biosecurity and containment measures rely on science-based disinfection protocols, where quaternary ammonium compounds (QACs) have been largely recommended [16,17].

QACs are cationic surfactants that penetrate the cell membranes of microorganisms (bacteria, fungi, and enveloped virus), destroying proteins and nucleic acids, causing cell lysis and death [18,19]. QACs are classified based on their R groups, nitrogen atoms number, their union to the carbon chain, and the presence of aromatic groups. These characteristics are essential for the antimicrobial activity both in terms of dosage and the target in the microorganisms. The evolution of QACs has been reflected as follows: first generation (single chain) Benzalkonium chloride, in which the alkyl group has an even number of alkyl chains C_12_ a C_18_; second generation (single chain): Benzalkonium was replaced by compounds with aromatic rings with hydrogen and chlorine groups and methyl and ethyl groups; third generation: blends of first and second generation, which have been found to be synergistic, with enhanced biocidal activity with relatively lower toxicity; fourth generation (twin chain): with stronger antimicrobial capacity compared to the single-chain QACs, improved tolerance to anionic surfactants, protein soil and water hardness salts, and lower toxicity; fifth generation: mixtures of selected twin chains; sixth generation: polymeric QACs; and seventh generation: Bis- QACs with polymeric QACs [18].

In addition to QACs, the efficacy of other disinfectants, such as those based on glutaraldehyde (GA), has been studied [14,20]. These products modify the alkyl radical from amino, hydroxyl, sulfhydryl and carboxyl groups of microorganisms affecting RNA, DNA, and protein synthesis [21]. GA disinfectant formulations have protein-fixing properties, establishing strong crosslinking with proteins on the microbial surface resulting on potent antimicrobial effects [22].

Both QACs and GA-based disinfectants are widely used as disinfectants in the industrial, biomedical, and, more recently, agricultural fields [23]. Previous studies have also confirmed the efficacy of QACs against *Foc* TR4 [14,20]. However, disinfectants may vary according to composition, blend, the concentration of active ingredients, exposure time, local conditions, soil type, among others. On the other side, the increasing demand for biosecurity measures to contain both agricultural and human diseases have raised the popularity of disinfectants, with a range of commercial brands becoming available. Most of these products were not tested for specific situations, such as disinfecting tools in the presence of soil, as is the case for *Foc* TR4. Therefore, before making any recommendation, the biocide efficacy of these products needs to be tested, simulating the conditions where and target against they will be used.

After almost two years of its official report in Colombia, *Foc* TR4 is still confined to La Guajira Department and is systematically monitored by the National Plant Protection Organization (ICA-Instituto Colombiano Agropecuario) to guarantee its containment and avoid its dissemination. So far, there is no scientific evidence that *Foc* TR4 could be eradicated. Aiming to verify the efficacy of the available commercial disinfectants in Colombia, in this study, the biocide effect of 10 QACs-based products and one GA were evaluated on a *Foc* TR4 strain isolated from La Guajira, Colombia [24].

## 2. Material and Methods

### 2.1. Inoculum Production

The *Fusarium oxysporum* f.sp. *cubense* TR4 strain 140038, isolated from symptomatic Cavendish banana from Dibulla, La Guajira state (Colombia) was provided by Instituto Agropecuario Colombiano (ICA). The strain was maintained in potato dextrose agar (PDA) at 27 °C.

To obtain micro and macroconidia, *Foc* TR4 was grown for seven days in PDA supplemented with chloramphenicol at a concentration of 50 µg/mL to prevent bacterial contamination at 27 °C. Once the strain was grown, the mycelium was collected using a sterile spreader and 10 mL of a 0.1% Tween 80 solution. After, the solution was filtered using a sterile miracloth (Millipore, Merck) to separate the spores from the rest of mycelia.

For chlamydospore production, a culture medium with soil substrate and plantain roots was used. The protocol was kindly provided by the National Banana Corporation of Costa Rica (CORBANA). Briefly, 100 g of healthy roots from plantain plants were macerated at maximum speed in a blender for one minute. Then, the liquid was separated from the root material with a miracloth. The material collected in the miracloth was dried in aluminum trays at 50 °C for 24 h. The following day, plantain roots were blended for a second time to have root powder. The root powder was then mixed in 500 mL Erlenmeyer with soil substrate in a 1:3 (plantain-root:soil) *v*/*v* relation. The resulting PRS (plantain-root-soil) culture medium was autoclaved.

An Erlenmeyer containing PRS culture medium was inoculated with 10 mL of a seven-days-old *Foc* TR4 culture grown in potato dextrose broth (PDB) and incubated during 21 days at 27 °C in darkness. Starting on day twenty-one, daily microscope observations to determine the presence of chlamydospores were carry out. Once observed, chlamydospores were isolated from PRS culture medium following the protocol described by Nguyen and colleagues [14]. Briefly, the medium/mycelium were washed with sterile distilled water using a needle syringe over a sterile miracloth to remove the microconidia. Then, the mycelium was transferred to a recipient on a shaker at 500 rpm for an hour, to break the mycelial clumps and release the chlamydospores. Next, the mycelium was filtered with a miracloth and the liquid bellow was centrifuged at 7000× *g* for 30 min. After centrifugation, the supernatant was eliminated, and the pellet was resuspended in 50% saccharose and centrifuged again (2 min, at 7000× *g*) to eliminate the soil. As soil particles were stock in the pellet, and the supernatant carries microconidia and chlamydospores, another centrifugation (30 min, at 7000× *g*) was carried out to eliminate the microconidia from the pellet. The supernatant with the chlamydospores was then centrifuged for one hour, and the isolated chlamydospores were collected. The pellet was resuspended in sterile distilled water, and chlamydospores again centrifuged (1 h, at 7000× *g*) to eliminate the saccharose residues. Chlamydospores were stored at 4 °C until used.

### 2.2. Evaluation of Disinfectants Efficacy on Foc TR4 Propagules in the Absence of Soil

A total of 10 commercial disinfectants were tested (Table 1). Based on previous reports of effective QACs doses [14], the concentration of all QACs-based disinfectants was adjusted to 1200 ppm. Sterile distilled water was used as a reference. Two exposure disinfection times (<1 and 15 min) were evaluated on conidia (macro and microconidia) and chlamydospores. Pathogen (*Foc* TR4 strain 140038) inoculum was adjusted to a final concentration of 1 × 10^6^ CFU.mL^−1^.

Aliquots of 100 µL of pathogen inoculum were added into 2 mL tubes containing 900 µL of the disinfectant solution vortexed to homogenize the mixture. After either <1 or 15 min, the spore/disinfectant solution was diluted with Tween 80 at 0.1% to a 1:10 ratio, plated on PDA, and incubated at 27 °C for seven days when the CFU.mL^−1^ were estimated. For CFU quantification, plates without disinfectant treatment with over 200 colonies (control) were usually counted by performing an additional 10-fold dilution step. After counting, to estimate the total number of CFU in the original culture, the total amount of colonies was multiplied by the dilution factor.

### 2.3. Evaluation of the Efficacy of Disinfectants on Foc TR4 Propagules in the Presence of Soil

Samples of soil (10.56% of organic matter) were sieved through a 2 mm soil sieve (No. 10). Then, 100 mg of soil was placed on 2 mL tubes and autoclaved three times. A 200 µL of inoculum suspension was prepared as described above, at a final concentration of 5 × 10^5^ spores.mL^−1^ added to the 100 mg of autoclaved soil and vortexed to homogenize the soil/pathogen mix. Then, the 2 mL tubes with the soil and pathogen spores were incubated at 27 °C for 24 h. After the incubation period, an 800 µL aliquot of each disinfectant was added to the 2 mL tubes to assure a final concentration of 1200 ppm for each QAC disinfectant. The tubes were briefly vortexed to ensure a proper mix of the disinfectant with the soil. Subsequently, disinfectants were allowed to act for <1 and 15 min, respectively. After the exposure times, a 300 µL aliquot of the mix solution was transferred into 15 mL falcon tubes containing 2700 µL of a 0.1% Tween 80 solution. Then, 1 mL of each treatment was placed in PDA containing Petri dishes. Plates were incubated at 27 °C for 7 days when the inhibition efficacy was evaluated. For GA-based disinfectant, four different concentrations (500, 800, 1200, and 2000 ppm) were tested as described previously.

### 2.4. Experimental Design and Statistical Analysis

For each treatment, three PDA plates (technical replicates) were used in a randomized design. To maximize the statistical reliability of the data, three biological replicates were carried out. The data were analyzed non-parametrically as the assumptions of the ANOVA (normality) were not fulfilled. The Kruskal–Wallis test was used to analyze the statistical differences between treatments, using the software Statistix version 8.0 with a probability value of *p* = 0.05.

The efficacy of each disinfectant was calculated using Equation (1): where *a* represents the value of CFU in the negative control, and *b* represents CFU in a disinfectant treatment [25]

(1) $$E=\left(\frac{a-b}{a}\right)*100$$

## 3. Results

The protocols used were efficient to both produce and purify *Foc* TR4 strain 140038 propagules, including chlamydospores, and subsequently perform studies on the efficacy of disinfectants with these structures.

### 3.1. Evaluation of Disinfectants Against Foc TR4 Propagules in the Absence of Soil

**Figure 1:**
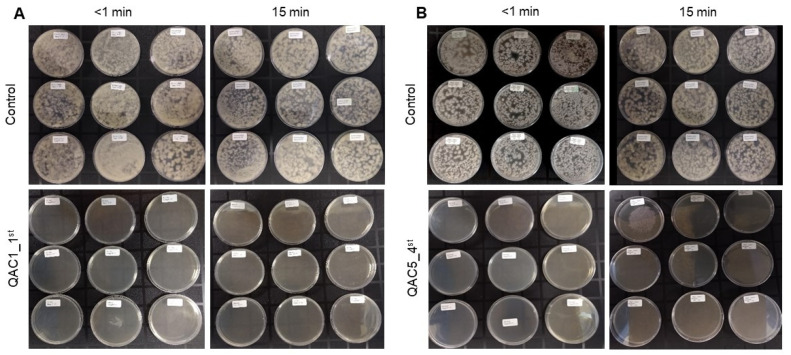
Representative image showing the efficacy of disinfectants based on quaternary ammonium compounds (QACs) against *Fusarium oxysporum* f. sp. *cubense,* tropical race 4 (*Foc* TR4) in the absence of soil at two exposure times (<1 and 15 min). (**A**) Macro- and microconidia of *Foc* TR4 with (QAC1_1st) and without (control) disinfectant treatment. (**B**) Chlamydospores of *Foc* TR4 with (QAC5_4th) and without (control) disinfectant treatment. Photographs taken by LFIG and S.L.C. Photographic records are from the Agricultural Microbiology Group, AGROSAVIA.

In the absence of soil, all QAC disinfectants (at 1200 ppm) showed 100% efficacy against macro, microconidia, and chlamydospores of *Foc* TR4, independently of the exposure time and evolution/generation (Figure 1, Supplemental Table S1).

### 3.2. Evaluation of Disinfectants Against Foc TR4 Propagules in the Presence of Soil

**Figure 2:**
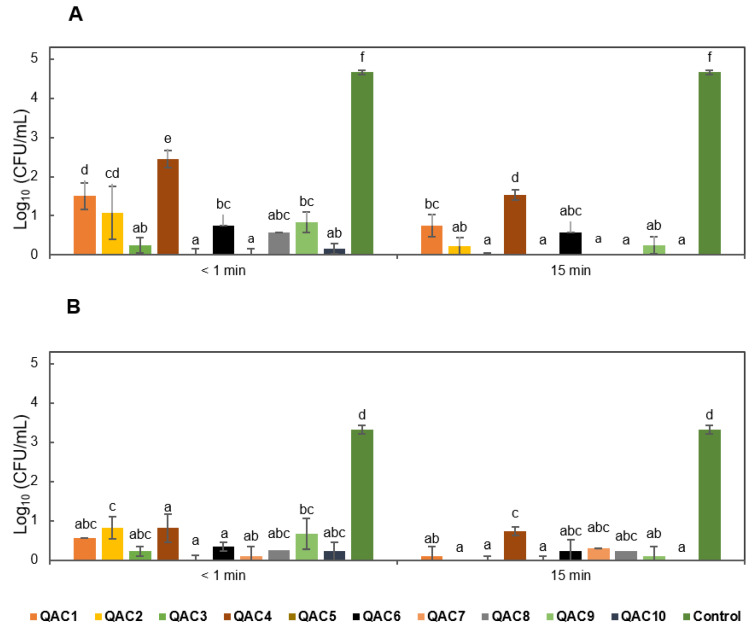
Efficacy of disinfectants based on quaternary ammonium compounds (QACs) against *Fusarium oxysporum* f. sp. *cubense*, tropical race 4 (*Foc* TR4) in the presence of soil. (**A**) Macro- and microconidia. (**B**) Chlamydospores. Bars with the same letter do not show significant differences among treatments using a Kruskal–Wallis test based on ranks (α = 0.05). Values of an independent biological replicate were taken to construct this figure; the three biological replicates are presented in Supplementary Figure S1.

All the QAC-based disinfectants reduced spore germination of *Foc* TR4 at both exposure times evaluated with inhibition values ranging from 80 to 100% (Figure 2). QAC5_4th showed 99–100% biocide activity against micro and macroconidia after both times (<1 and 15 min) of exposure (Figure 2A).

Six QAC-based disinfectants (QAC2_1st, QAC3_1st, QAC7_4th, QAC8_5th, QAC9_5th, and QAC10_5th) showed more than 95% biocide activity against micro and macroconidia of *Foc* TR4, but after 15 min of exposure. The other three disinfectants (QAC1_1st, QAC4_1st, and QAC6_4th) also reduced the spore viability, but in the range of 70-91%, allowing the growth of CFU of *Foc* TR4. Control plates (without disinfectant treatment) showed 100% viability of *Foc* TR4 conidia at both time points (Figure 2A and Supplementary Table S1).

Similar to the results obtained with the micro and macroconidia, QAC5_1st showed a 100% biocidal effect against chlamydospores in the presence of soil at both exposure times (Figure 2B). QAC3_1st and QAC8_5th were also 98–100% and 94–97% effective against *Foc* TR4 chlamydospores, respectively, across the two contact times. The remaining QAC disinfectants reduced chlamydospore viability after both <1 and 15 min of exposure but at lower levels (82–97%) of efficacy (Figure 2B). It is also important to note that four disinfectants (QAC1_1st–QAC4_1st) contain the same active ingredient: benzalkonium chloride; however, these products presented different efficacy levels against *Foc* TR4 in the presence of soil ranging from 55 to 98% for micro and macroconidia and from 82 to 100% for chlamydospores (Figure 2 and Supplementary Table S1).

**Figure 3:**
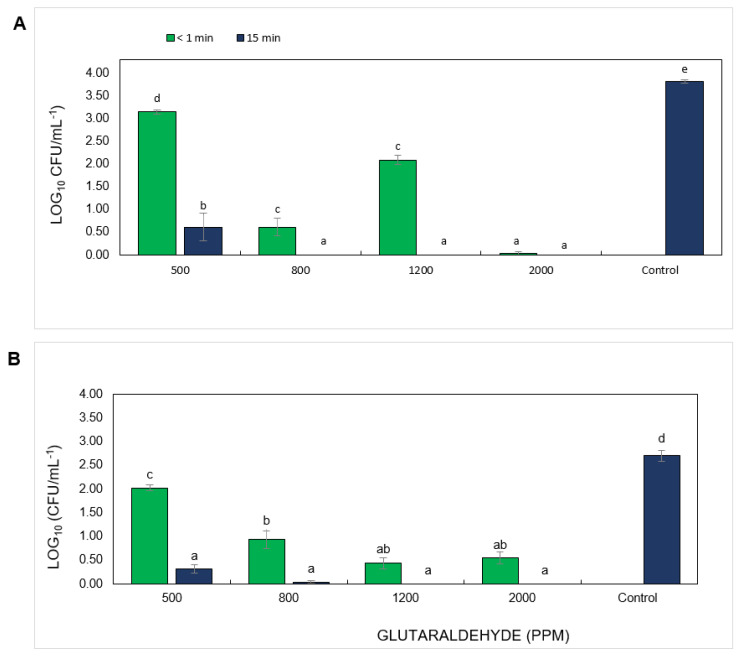
Efficacy of different concentrations of glutaraldehyde (GA)-based disinfectant *Fusarium oxysporum* f. sp. *cubense*, tropical race 4 (*Foc* TR4) in the presence of soil. (**A**) Macro- and microconidia. (**B**) Chlamydospores. Bars with the same letter do not show significant differences among treatments using a Kruskal–Wallis test based on ranks (α = 0.05).

**Figure 4:**
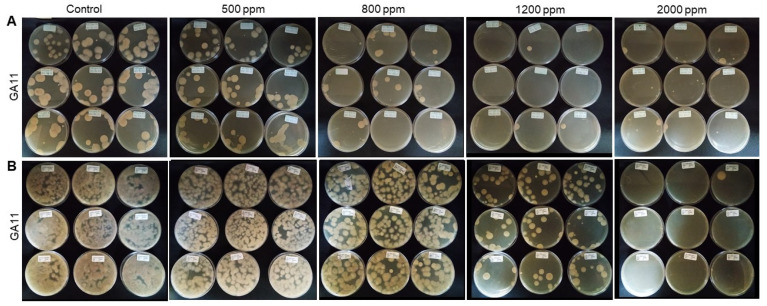
Representative image showing the efficacy of GA-based disinfectant against *Fusarium oxysporum* f. sp. *cubense*, tropical race 4 (*Foc* TR4) in the presence of soil after <1 min of contact. (**A**) Macro- and microconidia. (**B**) Chlamydospores. Four different concentrations (500, 800, 1200, and 2000 ppm) were evaluated. Photographs taken by LFIG and S.L.C. Photographic records are from the Agricultural Microbiology Group, AGROSAVIA.

In contrast to QACs, there were no recommendations or previous reports about the optimal exposure time, efficient concentrations, and suitable commercial products for on-farm use of GA-based disinfectants. For this reason, apart from 1200 ppm, three additional concentrations (500, 800, and 2000 ppm) were tested. The GA-based disinfectant tested did not eliminate all *Foc* TR4 propagules at <1 or 15 min of contact when used at 1200 ppm (Figure 3 and Figure 4). Even at a concentration of 2000 ppm, this product was not effective in reducing the survival of all propagules after >1 min of contact time (Figure 4). However, after 15 min of exposure, the concentrations of 1200 and 2000 ppm effectively prevented the germination of all *Foc* TR4 propagules (Figure 3 and Supplementary Table S2). Significant differences were found between treatments and doses, K_(8,89)_ = 74,9761, *p* = 0.05 (Figure 3).

## 4. Discussion

Exclusion is by far the best management practice for any plant disease, especially in the absence of resistant cultivars and for soil-borne pathogens, as are the cases of banana and *Foc* TR4. In such a scenario, and even when resistant varieties are available, establishing science-based biosecurity measures is essential [26]. Wherever *Foc* TR4 has arrived, growers have only sustained the production by implementing management strategies based on early detection of infected plants, eradication, but above all, having strict biosecurity protocols in place [27]. Farms must have proper disinfection areas for vehicles, equipment, boots, and personal hygiene facilities, among others [28]. However, the success of these biosecurity protocols strongly relies on quality-tested disinfection procedures, which include reliable information on the efficacy of available disinfectants in local conditions.

The efficacy of disinfectants against *Foc* TR4 has already been assessed [14,20]. Some studies have used fungicides of organic origin [29]. Others have assessed the effect of QACs for pathogen disinfection [14,20,30,31].

In this work, we evaluated the efficacy of 10 commercial quaternary ammonium compounds (QACs) products and one based on glutaraldehyde (GA) against *Foc* TR4 in the presence or absence of soil.

In the absence of soil, all QAC disinfectants (at 1200 ppm) showed 100% efficacy against macro-, microconidia, and chlamydospores of *Foc* TR4, even after less than one minute of exposure. Similar results with two QACs disinfectants showed 100% of biocide activity against *Foc* TR4 microconidia in the absence of soil but at different exposure times 5, 10, and 15 min [30].

In another study, the use QACs showed high efficacy against spores of the cotton pathogen *Fusarium oxysporum* f. sp. *vasifectum* [13]. However, similar to what we found in this study, the efficacy of QACs was reduced in the presence of soil [13]. Indeed, for eight out of 10 QACs-based disinfectants tested, in the presence of soil, the biocidal effect against *Foc* TR4 decreased depending on the product tested from 4 to 19% for conidia at shorter contact times (<1 min), and from 2 to 14% at longer contact times (15 min). For chlamydospores, the biocidal effect decreased from 2 to 13% for conidia at shorter contact times and from 2 to 7% at longer contact times (15 min). (Supplementary Table S1). However, QAC4_1st and QAC5_1th presented a different behavior, the presence of soil highly decreased the efficacy of QAC4_1st disinfectant, 45% at shorter exposure times for conidia, whereas efficacy of QAC5_1th disinfectant remained close to 100% in presence of soil across all contact times for both conidia and chlamydospores.

Organic matter (OM) present on soil samples contributes to diminishing the biocidal activity of disinfectants, including QACs and GA [14,32,33]. Nguyen and colleagues [14] evaluated the inhibitory effect on the disinfectant activity of two types of soil ferralsol (on *Foc* race 1) and kandosol (on *Foc* TR4). Both soil types are common in the American continent [34]. The OM content was not shown for these two soils used, but ferralsol soils usually have OM contents varying from 0.7 to 3%, and in some exceptions, they could reach up to 9% [35]. Soil samples (124) from banana plantations collected in La Guajira, Colombia, showed an average OM content of 1.80%. In our study, we used an Andosol soil with high organic matter contents (10.56%) to simulate what could happen in the field with such levels of OM content. These differences in OM content might explain the differences in the QACs efficacy obtained in our work and Nguyen and colleagues [14].

It has also been reported that OM may affect the effectiveness of GA-based disinfectants, GPC8 a GA-based commercial disinfectant, was only effective in low or no organic matter suspensions against porcine rotavirus [36]. Two mechanisms have been described to explain the fact that the organic matter has a negative effect on the QACs biocidal activity (i) one is that the organic matter might act as a physical barrier protecting the microorganisms, and (ii) the other is that the organic matter interacts with the disinfectant molecule due to its cationic charge, affecting the availability of the disinfectant to be in contact with the microorganism [37].

Meldrum and colleagues [28] evaluated *Foc* TR4 microconidia germination after four exposure times 30 s, 1, 5, and 15 min, to a QAC of 4th generation and a sodium hypochlorite-based disinfectant; and there was a 100% inhibition at all four time points selected. However, a commercial detergent used for agricultural machinery cleaning showed less than 60% inhibition at any time point. Interestingly, the product based on sodium hypochlorite lost its inhibiting capacity after one month at temperatures ranging from 22–58 °C, and constant light exposure, while QAC-based disinfectant maintained its inhibition efficacy of 100% at the same harsh conditions for the same exposure time, demonstrating the stability of QACs [20].

Recently, the efficacy of QACs and other disinfectants was evaluated on *Foc* TR4 in the presence of soil (0.05 g·mL^−1^), showing 100% biocidal efficacy at the exposure times evaluated from 30 s to 24 h, as a concentration of 1200 ppm [14]. Additionally, the efficacy of GA-based products alone or in combination with QACs against pathogenic microorganisms has also been assessed [23,33,38].

In our study, we tested one GA-based disinfectant, which was not 100% effective at 1200 ppm and even at 2000 ppm did not effectively inhibit all *Foc* TR4 chlamydospores after <1 min of exposure. However, this product showed 100% efficacy against *Foc* TR4 after 15 min, suggesting that the exposure time plays an important role for this product at 1200–2000 ppm.

Regarding the use of QACs as a wide strategy for disinfection in banana farms, it is important to consider that some microorganisms, such as the bacteria *Xanthomonas* spp. and *Pseudomonas* spp. can degrade QACs by breaking the Calkyl-N bond [39,40]. Therefore, site-specific studies might be needed. In addition, most of the studies on disinfectants’ efficacy in the presence of soil have used sterile soils, which might have an impact on the outcome of the biocidal efficacy. Studies assessing the biocidal effect of QACs using non-sterilized soil samples from banana plantations should be considered. Currently, we are testing in the field the efficacy of commercial disinfectants for the treatment of footbaths, drive vehicles, irrigation water, and checkpoints established by ICA in La Guajira, Colombia.

QACs disinfectants are among the most widely used as they are not corrosive, are highly stable, exhibit high biocidal efficacy against microorganisms, and present low toxicity risks for humans and animals [18]. However, our results show that in the presence of soil, its efficacy against *Foc* TR4 was reduced. Therefore, biosecurity measures should not only include the use of disinfectants but also consider that soil can significantly hamper their effectiveness. In that sense, extensive washing to remove soil from vehicles, tools, boots, among others, prior to disinfection is essential.

The incursion of *Foc* TR4 in Colombia, which is so far restricted to La Guajira, raised alarms up, not only for other pathogen-free banana-producing regions in the country but for all banana-producing countries in Latin America and the Caribbean (LAC). Keeping *Foc* TR4 restricted to La Guajira will not only safeguard the Colombian banana industry valued at 858 million USD [41,42] but the livelihoods of more than 12 million people who depend on this industry in the Latin American and the Caribbean region [16].

While having the *Foc* TR4 already in the region doubtless increased the dissemination risks, LAC countries, including Colombia, must be aware that new incursions could eventually occur from other continents. In most countries where *Foc* TR4 is present, the disease is already considered endemic, with high risks of the pathogen being disseminated. LAC countries must implement strict biosecurity protocols from borders to farm gates considering *Foc* TR4 as a global threat.

## 5. Conclusions

In this work, we provide scientific evidence to recommend locally available disinfectants which can limit the spread of *Foc* TR4. However, for most of the products, complete effectiveness against *Foc* TR4 propagules was not achieved in the presence of soil in a final concentration of 1200 ppm. Thus, it is crucial to complement the use of disinfectants with additional on-farm biosecurity procedures, including complete removal of soil from equipment and tools before disinfection. To select higher concentrations of QACs or GA-based disinfectants for on-farm decontamination procedures, other factors such as product cost, exposure time, type of surface to be decontaminated (soaking boots or tools), corrosiveness, longevity, and environmental impact should be considered.

## Figures and Tables

**Table 1:** Disinfectants evaluated and their chemical characteristics (active ingredient and quaternary ammonium compounds generation).

Disinfectant (Treatments) ^Ψ^	Active Ingredient *	QAC Generation
QAC1_1st	Benzalkonium chloride	1st
QAC2_1st	Benzalkonium chloride	1st
QAC3_1st	Benzalkonium chloride	1st
QAC4_1st	Benzalkonium chloride	1st
QAC5_4th	Dimethyl ammonium chloride	4th
QAC6_4th	Didecyldimethylammonium chloride	4th
QAC7_4th	Benzalkonium chloride+Didecyldimethylammonium chloride	4th
QAC8_5th	Di(octyl/decyl) chloride + dimethyl ammonium + benzalkonium chloride	5th
QAC9_5th	Quaternary ammonium	5th
QAC10_5th	Quaternary ammonium	5th
GA11 ^a^	Glutaraldehyde	-

^a^ GA: glutaraldehyde. Studies with GA were performed only in the presence of soil. * Together with the National Plant Protection Organization (ICA-Instituto Colombiano Agropecuario), a list of the most popular QACs-based commercial products available in the country was constructed to be evaluated against *Foc* TR4 isolated from La Guajira, Colombia. Treatments with the same active ingredient correspond to different commercial trademarks. ^Ψ^ Codes were used instead of disinfectant trade names to attend to internal rules of the Intellectual Property Department of AGROSAVIA.

## Data Availability

The data that supports the findings of this study are available in the supplementary information of this article. Any additional data will be available on request to the corresponding author (msoto@agrosavia.co).

## Supplementary Materials

The following are available online at https://www.mdpi.com/article/10.3390/jof7040297/s1, Figure S1: Evaluation of disinfectants efficacy in the presence of soil on micro and macroconidia. Concentration of *Foc* TR4 CFU after exposing (A) micro and macroconidia, (B) chlamydospores at two contact times (<1 min and 15 min). Bars with the same letter do not show significant differences among treatments using a Kruskal Wallis test based on ranks (α = 0.05). R1, R2 and R3 indicate biological replicates. For micro and macroconidia, comparing the results from three biological replicates of the same experiment, significant differences were observed with respect to the control, K(22,68) = 57.6044, *P* = 0.000050176; K(22,68) = 52.3458, P= 0.000280017, K(22,68) = 41.6188, *P* = 0.00694946 for biological replicates R1, R2 and R3, respectively. For the treatments of disinfectants on chlamydospores in the presence of soil, for the three biological replicates were also observed significant differences with respect to the control, K(22,67) = 40.62 P = 0.00913933; K(22,67) = 39.55, *P* = 0.0121891, K(22,67) = 36.33, *P* = 0.0279374 for biological replicates R1, R2 and R3, respectively; Table S1: Efficacy of Quaternary Ammonium Compounds (QACs) disinfectant against *Fusarium oxysporum* f. sp. *cubense*, tropical race 4; Table S2. Efficacy of different concentrations of a Glutaraldehyde-based disinfectant against *Fusarium oxysporum f.* sp. cubense, tropical race 4 in presence of soil.
