# Supplementary material for: Efficacy of Disinfectants against Fusarium oxysporum f. sp. cubense Tropical Race 4 Isolated from La Guajira, Colombia
Source: J Fungi (Basel). 2021 Apr 15;7(4):297. doi: 10.3390/jof7040297 (PMC8071173; doi:10.3390/jof7040297)
Supplement: Supplementary file 1 [file jof-07-00297-s001.zip › jof-1187293-supplementary.pdf]

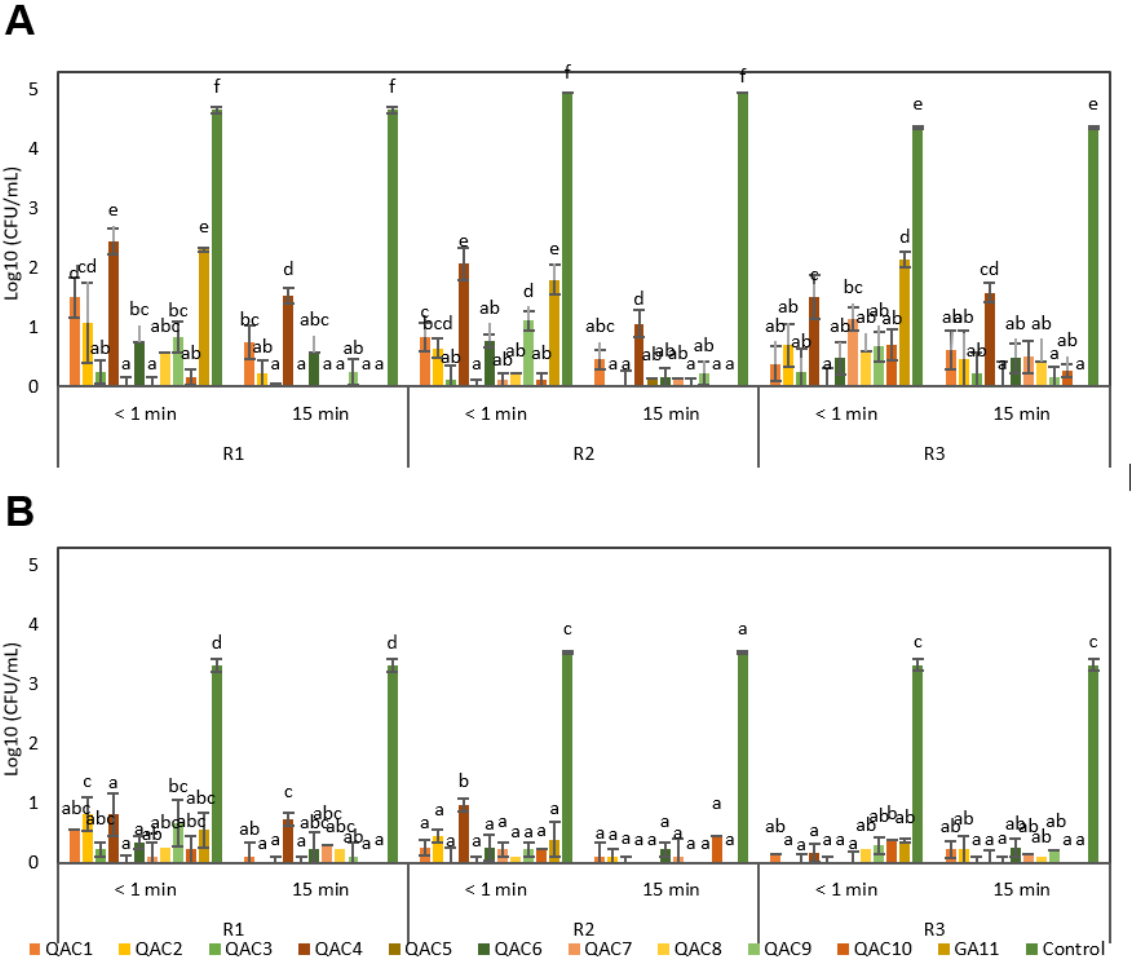

Figure S1. Evaluation of disinfectants efficacy in the presence of soil on micro and macroconidia. Concentration of *Foc* TR4 CFU after exposing (A) micro and macroconidia, (B) chlamydospores at two contact times (<1 min and 15 min). Bars with the same letter do not show significant differences among treatments using a Kruskal Wallis test based on ranks (α = 0.05). R1, R2 and R3 indicate biological replicates. For micro and macroconidia, comparing the results from three biological replicates of the same experiment, significant differences were observed with respect to the control, $K_{(22,68)}$= 57.6044, *P= 0.000050176;* $K_{(22,68)}$= 52.3458, *P= 0.000280017*, $K_{(22,68)}$= 41.6188, *P= 0.00694946* for biological replicates R1, R2 and R3, respectively. For the treatments of disinfectants on chlamydospores in the presence of soil, for the three biological replicates were also observed significant differences with respect to the control, $K_{(22,67)}$= 40.62 *P= 0.00913933*; $K_{(22,67)}$= 39.55, *P=0.0121891*, $K_{(22,67)}$= 36.33, *P=0.0279374* for biological replicates R1, R2 and R3, respectively.

Table S1. Efficacy of Quaternary Ammonium Compounds (QACs) disinfectant against *Fusarium oxysporum* f. sp. *cubense*, tropical race 4.

Treatment	Time	Conidia		Chlamydospores	
		Efficacy (%)		Efficacy (%)	
		Without soil	With soil	Without soil	With soil
**QAC1_1st**	< 1 min	100.0 ± 0.0	80.6 ± 14.0	100.0 ± 0.0	90.4 ± 9.9
	15 min	100.0 ± 0.0	86.9 ± 8.6	100.0 ± 0.0	95.5 ± 5.3
**QAC2_1st**	< 1 min	100.0 ± 0.0	82.7 ± 15.0	100.0 ± 0.0	87.3 ± 13.3
	15 min	100.0 ± 0.0	95.0 ± 10.7	100.0 ± 0.0	96.7 ± 7.1
**QAC3_1th**	< 1 min	100.0 ± 0.0	95.5 ± 5.9	100.0 ± 0.0	97.8 ± 6.7
	15 min	100.0 ± 0.0	98.4 ± 3.2	100.0 ± 0.0	100 ± 0.0
**QAC4_1st**	< 1 min	100.0 ± 0.0	55.2 ± 9.8	100.0 ± 0.0	82.3 ± 14.9
	15 min	100.0 ± 0.0	70.1 ± 8.3	100.0 ± 0.0	92.6 ± 12.5
**QAC5_4th**	< 1 min	100.0 ± 0.0	100.0 ± 0.0	100.0 ± 0.0	100 ± 0.0
	15 min	100.0 ± 0.0	99.0 ± 3.1	100.0 ± 0.0	100 ± 0.0
**QAC6_4th**	< 1 min	100.0 ± 0.0	85.7 ± 9.8	100.0 ± 0.0	94.1 ± 5.7
	15 min	100.0 ± 0.0	91.4 ± 9.5	100.0 ± 0.0	93.0 ± 8.9
**QAC7_4th**	< 1 min	100.0 ± 0.0	91.1 ± 13.0	100.0 ± 0.0	96.7 ± 7.1
	15 min	100.0 ± 0.0	95.4 ± 7.3	100.0 ± 0.0	94.4 ± 9.5
**QAC8_5th**	< 1 min	100.0 ± 0.0	90.0 ± 8.0	100.0 ± 0.0	94.1 ± 5.7
	15 min	100.0 ± 0.0	96.9 ± 9.2	100.0 ± 0.0	96.7 ± 5.0
**QAC9_5th**	< 1 min	100.0 ± 0.0	81.3 ± 10.2	100.0 ± 0.0	88.2 ± 12.3
	15 min	100.0 ± 0.0	95.4 ± 5.7	100.0 ± 0.0	96.8 ± 6.6
**QAC10_5th**	< 1 min	100.0 ± 0.0	93.0 ± 8.0	100.0 ± 0.0	91.7 ± 8.7
	15 min	100.0 ± 0.0	98.0 ± 5.9	100.0 ± 0.0	95.5 ± 10.1

± Indicates standard deviation (*n*=9)

Table S2. Efficacy of different concentrations of a Glutaraldehyde-based disinfectant against *Fusarium oxysporum* f. sp. *cubense*, tropical race 4 in presence of soil.

Treatment	Time	Efficacy (%)	
		Conidia	Chlamydospores
500 ppm	< 1 min	17.1 ± 2.6	37.8 ± 5.7
	15 min	83.9 ± 24.7	90.5 ± 8.1
800 ppm	< 1 min	83.9 ± 24.7	71.4 ± 17.8
	15 min	100.0 ± 0.0	98.9 ± 3.4
1200 ppm	< 1 min	45.0 ± 8.7	98.9 ± 3.4
	15 min	100.0 ± 0.0	100.0 ± 0.0
2000 ppm	< 1 min	99.0 ± 2.9	85.3 ± 17.7
	15 min	100.0 ± 0.0	100.0 ± 0.0

± Indicates standard deviation (*n*=9)
